# The Role of Explanations in AI-Generated Alerts: Qualitative Study of Clinical Views on Explainable AI in Predictive Tools

**DOI:** 10.2196/81460

**Published:** 2026-05-01

**Authors:** Jessica Rahman, Alana Delaforce, DanaKai Bradford, Jane Li, Farah Magrabi, David Cook, Aida Brankovic

**Affiliations:** 1 Australian e-Health Research Centre, Commonwealth Scientific and Industrial Research Organisation (CSIRO), 160 Hawkesbury Road, Sydney, New South Wales, 2145, Australia, 61 2 9325 3016; 2 Australian e-Health Research Centre, Commonwealth Scientific and Industrial Research Organisation (CSIRO), Brisbane, Queensland, Australia; 3 School of Nursing and Midwifery, Griffith University, Brisbane, Australia; 4 Australian Institute of Health Innovation, Macquarie University, Sydney, Australia; 5 Intensive Care Unit, Princess Alexandra Hospital, Brisbane, Australia

**Keywords:** artificial intelligence, explainable artificial intelligence, explainable AI, disagreement in XAI, clinical decision support, qualitative research, human factors

## Abstract

**Background:**

Artificial intelligence (AI)–driven clinical decision support (CDS) tools offer promising solutions for health care delivery by optimizing resource allocation, detecting deterioration, and enabling early interventions. However, adoption remains limited due to insufficient validation and a lack of transparency and trust. Explainable AI (XAI) seeks to improve user understanding of AI outputs; however, how clinicians interpret and integrate these explanations into their decision-making remains underexplored. Furthermore, discrepancies in explanations, known as the “disagreement problem,” can undermine trust and, at worst, lead to poor clinical decisions.

**Objective:**

This study examines clinicians’ perspectives on the role and value of explainability in AI-driven CDS tools within Australian critical care settings and the impact of discrepancies in AI-generated explanations on clinical decision-making.

**Methods:**

Qualitative data were collected using semistructured interviews with 14 clinical experts, incorporating scenario-based exercises, and were analyzed using inductive thematic analysis.

**Results:**

Clinicians valued explainability, particularly in complex or unfamiliar situations, when explanations were clear, plausible, and actionable. Trust and perceived usefulness extended beyond explanation quality, encompassing factors such as system accuracy, alignment with clinicians’ reasoning, workflow integration, and perceived reliability. Discrepancies in explanations generated by different XAI methods were not a major concern, provided that the AI-generated predictive alerts were accurate.

**Conclusions:**

This study provides design recommendations for developing trustworthy, user-centric CDS tools that incorporate XAI. Findings highlight that explainability is critical for establishing initial trust in AI-driven tools by supporting perceived usefulness, but its importance diminishes over time and with user expertise and familiarity, as learned usefulness takes precedence. Recommendations highlight the importance of aligning the design and implementation of AI tools with clinicians’ needs to enhance trust, mitigate risks, and promote successful adoption for improved patient outcomes.

## Introduction

Artificial intelligence (AI)–driven clinical decision support (CDS) systems are emerging as powerful tools in health care, leveraging large-scale medical data to identify patterns and generate insights that help clinicians deliver more informed and effective care [1-3]. Beyond supporting clinical decision-making, these systems have the potential to optimize resource allocation and streamline workflows by processing large volumes of electronic health data and deploying scalable, cost-effective algorithms that enhance efficiency while minimizing additional resource burdens [4-8]. Furthermore, CDS tools can detect subtle patterns of deterioration and offer predictive insights to help clinicians intervene earlier [9,10]. However, despite the promise of AI in health care, these tools have not been widely adopted for several reasons. One significant barrier is the lack of real-world testing and validation [11-13]. While early evidence suggests that AI-driven tools hold promise beyond controlled academic settings, their effectiveness in dynamic, real-world clinical environments remains underexplored, and further research is needed to understand the factors influencing their adoption, including patient heterogeneity, system integration, and workflow compatibility [13,14].

Recent research on deployed decision support tools also highlights the lack of human factors consideration as a key driver behind their limited adoption in clinical settings [1,15-19]. This includes a lack of attention to how clinicians interact with technology, usability issues, and how well the tool fits into existing workflows [1,16-19]. Another major concern is the lack of explainability and transparency in many AI-driven CDS tools [20,21]. Clinicians are understandably hesitant to rely on tools they do not fully understand, especially in high-stakes environments such as hospitals [2,22]. When an AI-driven CDS system provides a recommendation or prediction, it is crucial for health care providers to understand the reasoning behind it. Without clear and understandable explanations, there is a risk that clinicians will not trust the tools, regardless of their accuracy [23].

Without adequate transparency, there is also a risk that clinicians may overrely on these tools, potentially leading to harmful outcomes [24-27]. This trust gap between health care providers and AI-driven CDS tools is a significant obstacle to widespread adoption [2,28]. Effective implementation of AI-driven CDS systems requires careful trust calibration, ensuring that clinicians develop an appropriate level of trust aligned with the system’s real-world performance, to support safe and effective use in practice [27,29].

To support trust calibration of AI-driven CDS tools, explainable AI (XAI) methods have been designed to provide transparency by not only presenting the predictions but also revealing the underlying rationales that drive those predictions [27,30-33]. By allowing medical professionals to see the logic behind the AI’s decision, XAI aims to bridge the gap between the complex algorithms used in machine learning and the human need for clarity and accountability in clinical practice. For tasks such as classification and regression, post hoc explanation methods are increasingly being used to shed light on the behavior of complex models after training [15,34,35]. Rather than altering the model, these methods aim to reveal how it makes decisions by producing clinically informative approximations or visual representations. However, recent findings in responsible AI have highlighted significant challenges in creating robust, user-centric, and context-sensitive explanations that can be trusted and easily comprehended [36,37].

While much of the existing literature has focused on the technical reliability or consistency of XAI methods, less attention has been paid to how clinicians interpret, evaluate, and use these explanations in practice. Understanding these human-centered aspects of explainability is critical for ensuring that AI-driven CDS tools support, rather than disrupt, clinical workflows and reasoning. One key challenge is that post hoc explanations generated by models can be overly sensitive to small input changes or offer convincing justifications for incorrect predictions [26,38]. Research has shown that when these explanations are presented alongside model outputs, they can unintentionally foster increased trust in the model, even if that trust is not justified, and lead to potentially harmful decisions [33,39-41]. Another challenge is the “disagreement problem,” which refers to discrepancies among state-of-the-art methods in the explanations they provide, as revealed by recent research [37,42-46].

The disagreement issue arises when different XAI techniques produce conflicting explanations, even when applied to the same input and underlying model prediction. While several studies have shown that AI-generated explanations can increase clinicians’ likelihood of accepting recommendations from CDS tools [1,47-50], they often rely on the implicit assumption that a correct model prediction is accompanied by a valid explanation. Previous studies have examined the impact of incorrect explanations on users when the AI’s prediction is correct [51]. However, they often overlook the fact that different XAI methods can produce conflicting explanations for the same prediction. Moreover, recent research has shown that such explanations can inflate users’ trust in the model even when that trust is not warranted [26,33,39,40]. Despite these concerns, little is known about how clinicians understand and respond to such discrepancies. This gap not only limits our understanding of explanation reliability in high-stakes settings but also complicates the task of selecting appropriate XAI methods for use in CDS tools.

Building on these insights, this study explores clinicians’ perspectives on the role and value of explainability in AI-driven CDS tools, focusing on how explanations are interpreted, trusted, and incorporated into clinical decision-making through qualitative interviews with clinical experts. It also explores how discrepancies between different explanation methods affect clinical judgments through a scenario-based approach. Additionally, we seek to identify key considerations for effectively integrating explainability into AI-driven CDS tools. Our study is focused on Australian health care settings, where there is a growing need for exploration of AI-driven CDS tools [52]; yet, limited empirical evidence exists regarding their acceptance and impact in real-world clinical practice. This study seeks to contribute to this area by exploring 3 key research questions (RQs) listed below:

- RQ1: How does explainability influence clinicians’ use, trust, and perceived usefulness of AI-driven CDS tools?
- RQ2: How do clinicians interpret and respond when different explainability methods provide differing explanation factors, despite the AI prediction being correct?
- RQ3: What key design considerations can support the trustworthy and user-centric integration of AI-driven CDS tools with explainability as a key component?

## Methods

### Study Design

Semistructured interviews were conducted with clinical experts to explore their views on XAI and the discrepancies observed between different XAI methods. During the interviews, participants were given a scenario-based exercise using an AI-based predictive CDS tool to assess how discrepancies in explanations influenced decision-making.

### Participant Recruitment

The study participants comprised clinical experts who were potential users of CDS tools designed for use in the intensive care unit (ICU) environment. Recruitment began by reaching out to current and former clinical collaborators to identify suitable participants. A purposive, snowball sampling method was used, allowing the identification of additional stakeholders as the research progressed [53]. A recruitment flyer was prepared and emailed to all potential participants. After participants signed the consent form, they scheduled an interview time using an online scheduler. Recruitment and data collection were conducted concurrently with data analysis, continuing until data saturation was reached [54].

### Data Collection

Semistructured interviews were conducted from February to July 2024. The interview was divided into 3 main segments, each containing multiple open-ended questions. The questions for the first 2 segments were adapted from the study by Schwartz et al [55]. The first segment focused on understanding the participants’ clinical experience and their general views on CDS and XAI. The second segment explored participants’ thoughts on factors influencing their trust in CDS tools and how XAI contributes to it. In the final segment, participants were presented with 2 scenarios based on an AI-based predictive CDS and explainability modules developed in a previous study using electronic medical record data [36]. Detailed descriptions of these scenarios are provided in the “Scenarios” subheading. A pilot interview was conducted with 1 participant, leading to further refinement of the questions and interview duration. The interview guide is available in Multimedia Appendix 1. Interviews were conducted online via Microsoft Teams and recorded using the platform’s built-in feature. Four team members (JR, AD, JL, and DB) with doctoral qualifications and expertise in qualitative research conducted the interviews, with at least 2 present at each session. Each session lasted between 45 and 60 minutes. Notes were taken during the interviews. All audio recordings were transcribed by a professional transcription service and deidentified upon return.

### Scenarios

**Figure 1.**
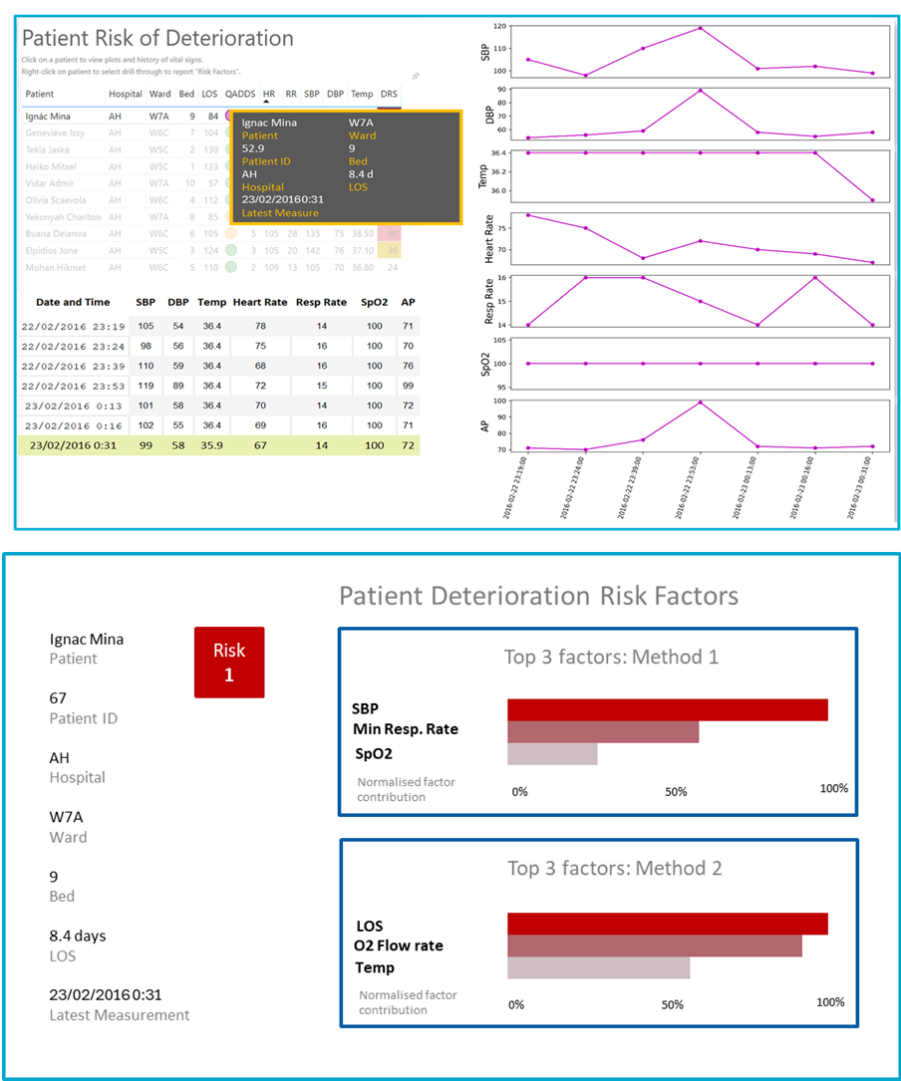
The first part of the clinical decision support (CDS) tool “(A)” showed a list of patients at risk of deterioration and recorded values of vital signs for selected patients in the last 24 hours, alongside the basic demographics. The second part of the tool “(B)” presents additional available information corresponding to the patient regarding the predicted deterioration risk. In addition to basic demographics about the patient, 3 main factors rationalizing the model prediction were presented, obtained using 2 different methods. The factors were ordered based on their normalized contribution in descending order. Both methods correctly predicted patient deterioration. The main contributor obtained with Method 1 matched the vital sign that triggered the alarm, unlike any of the contributors obtained with Method 2. AH: anonymized hospital identification; AP: arterial pressure; DBP: diastolic blood pressure; DRS: deterioration risk score; HR: heart rate; ID: identification; LOS: length of stay; Min: minimum; QADDS: Queensland Adult Deterioration Detection System; Resp: respiratory; RR: respiratory rate; SBP: systolic blood pressure; SPO2: oxygen saturation; Temp: temperature.

The CDS tool selected as the use case for this study was an algorithm that predicts the activation of an early warning score system, providing up to 8 hours of warning of potential patient deterioration. The algorithm was developed and validated using real-world data from a tertiary hospital in Queensland, Australia, and further methodological and technical details are available in prior publications [36,56]. The scenarios included encounter records, extracts with primary and additional diagnosis Systematized Nomenclature of Medicine codes, extracts indicating deteriorating alerts (red and yellow flags), and vital signs data. Explanations were generated by several post hoc explainers using real examples from the model’s training and testing datasets. The selected use cases were constructed to demonstrate instances in which the model’s predictions were accurate, while the corresponding explanations varied in their degree of correctness. This approach aimed to assess how domain experts not only see the explanations but also interpret and respond to discrepancies between model accuracy and explanation fidelity and to ensure that correct model predictions would not be disregarded solely due to imperfections in the accompanying explanations. Information was presented as a prototype of the tool planned for pilot implementation (Figures1  2). Further information on patient diagnoses and procedures was presented to the participants when the scenarios were shown, to provide contextual information about the considered cases and to realistically simulate the clinical scenarios. These details are provided in Multimedia Appendix 2.

**Figure 2.**
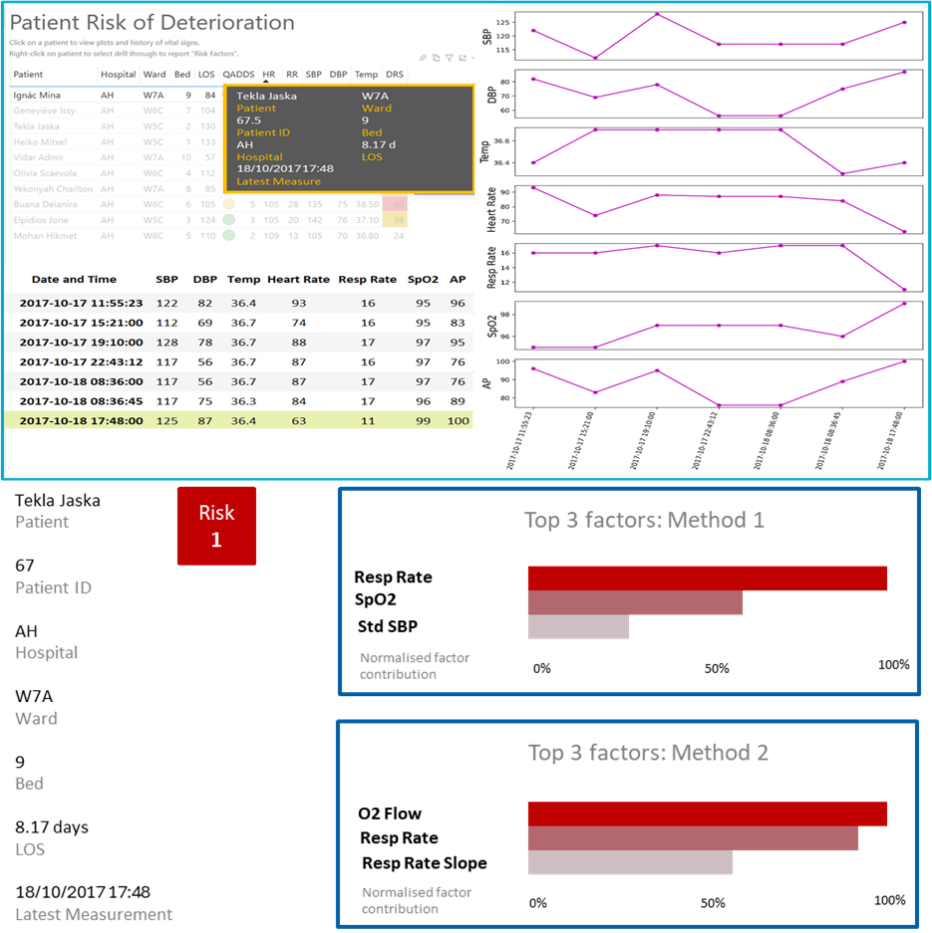
(A) and (B) illustrate results for a different patient. In this case, both predictive methods accurately identified patient deterioration, and both explainable artificial intelligence (XAI) techniques recognized the same contributing factor as indicated by the vital sign that triggered the alarm, but with differing ranks of importance. AH: anonymized hospital identification; AP: arterial pressure; DBP: diastolic blood pressure; DRS: deterioration risk score; HR: heart rate; ID: identification; LOS: length of stay; Min: minimum; QADDS: Queensland Adult Deterioration Detection System; Resp: respiratory; RR: respiratory rate; SBP: systolic blood pressure; SPO2: oxygen saturation; Temp: temperature.

### Data Analysis

The analysis aimed to uncover domains related to what influences trust in XAI and how discrepancies affect it. Due to the lack of suitable existing frameworks that adequately addressed the issues discussed by participants for deductive analysis, an inductive open coding approach using grounded theory [57] was used, allowing themes to emerge organically from the data without any preconceived categories or theoretical framework. The data analysis was conducted using Microsoft Excel. The COREQ (Consolidated Criteria for Reporting Qualitative Research) checklist is provided in Checklist 1.

Initial coding of each interview transcript was carried out by a qualitative researcher (JR). The coding was then sent to a second qualitative researcher (AD) for agreement checking. Consensus discussions were held, and all disagreements were resolved between the 2 researchers. Using the inductive approach, an initial codebook was developed after coding the first transcript. This codebook guided the coding of subsequent transcripts and was iteratively reviewed and updated following each consensus discussion. After completing the initial coding, the codes were grouped into broader domains. For each domain, the 2 qualitative researchers conducted a consistency check to refine the constructs and ensure all data were coded consistently. At this stage, 1 researcher (JR) also inductively summarized each text artifact into a statement that captured its meaning, referred to as subconstructs. A consensus discussion was then conducted with the second researcher (AD), using the same method as the initial coding discussions, to further refine and condense the statements where needed. This iterative process of consistency checking at each stage enabled the study team to triangulate the data, incorporating multiple perspectives to strengthen confidence in the study’s findings. After refinement, the preliminary findings were emailed to all interview participants, following their agreement to participate in a “member checking” process. Participants were asked to provide feedback on whether the preliminary findings captured the most important issues and on the clarity of the phrasing used for constructs and statements. Responses were received from 57% (8/14) of participants, comprising affirmative and/or constructive feedback. This feedback informed further refinement and changes to the final set of domains and constructs.

### Ethical Considerations

Ethics approval for this study was obtained from the Commonwealth Scientific and Industrial Research Organisation (CSIRO) Health and Medical Research Ethics Committee (reference no 2023_074_LR). A privacy review of the study was also conducted by a legal counsel at CSIRO. A recruitment flyer, which included a link to the online participant information sheet and digital consent form, was prepared and emailed to all potential participants. The digital consent form was hosted in the CSIRO’s secure REDCap (Research Electronic Data Capture; Vanderbilt University) application. The information sheet provided details about the study’s purpose, privacy considerations, potential risks and benefits, and how the data would be collected and used. Upon completing their participation, participants were provided with an AUD $50 (approximately US $35) gift card.

## Results

### Participant Demographics

Clinical experts (N=14) took part in the interviews (Table 1). Experience levels were categorized as follows: “Junior”= <10 years of experience, “Mid”= 10‐20 years, and “Senior”= >20 years. Overall, there was equal participation from both doctors (7/14) and nurses (7/14) working in the ICU environment, with 64% (9/14) in the adult, 29% in the pediatric (4/14), and 7% (1/14) in the neonatal ICU. Of the participants, 50% (7/14) were identified as senior clinical experts (4 females), whereas 29% (4/14, 2 females) and 21% (3/14, 2 females) were identified as mid and junior level, respectively. Participants were based in 5 states and territories of Australia, comprising the Australian Capital Territory, New South Wales, Victoria, Queensland, and Western Australia.

**Table 1.** Demographics of the interview participants.

Category and subcategory	Participants, n (%)
Role	
Doctor	7 (50)
Nurse	7 (50)
Experience	
Junior	3 (21.4)
Mid	4 (28.6)
Senior	7 (50)
Sex	
Male	6 (42.9)
Female	8 (57.1)
ICU^a^ environment	
Adult	9 (64.3)
Pediatric	4 (28.6)

a ICU: intensive care unit.

Participants were also asked about their current use of CDS systems, which could be AI-powered or not. The majority (n=11) of participants indicated they had experience with CDS tools; however, they were not AI-driven. Crude rule-based scoring systems were still prevalent, and while they provided a framework for assessing risk, they lacked the maturity needed for real-time individualized care adjustments. Three participants indicated that they did not have any experience with computer-based CDS tools because their current systems were paper-based and relied on manual charting of patient observations. All participants recognized the potential of AI to address clinical challenges, and many felt that the systems currently in use were relatively simple and would benefit from further development and integration into everyday clinical practice.

### Findings

#### Overview

An inductive thematic analysis using open coding identified 6 overarching domains. Of these, 3 focused on AI-driven CDS with XAI as a key component and are therefore reported here: (1) explainability and actionability, (2) trust, and (3) design recommendations for AI-driven CDS with XAI. The constructs and subconstructs associated with these 3 domains are presented in Table 2. The number of participants refers to the number of participants who raised a construct at least once across interviews. For example, a participant may have mentioned an issue with high frequency in their interview, but it was only counted once to avoid potentially skewed data. Exemplar quotes for each of the subconstructs are provided. The remaining 3 domains identified broader use, concerns, and limitations surrounding AI-powered CDS tools, as well as implementation considerations for successful adoption. These additional findings are presented in Multimedia Appendix 3.

**Table 2.** Constructs and subconstructs of the 3 key domains identified in the qualitative analysis.

Domain, construct, and subconstruct	Exemplar quote
Explainability and actionability	
Need and usefulness of explainability (n=7)	
Complexity of both the patient condition and decisions to be made (n=6).	*“*…there may be less need for complex explanation when it’s saying put patient X in bed X, because they’re likely to move by this date as opposed to if I’m trying to diagnose a pneumonia which might be confused for a COPD type thing…” (P11, Nurse, Mid, Pediatric)
Clinicians’ experience level (n=2).	*“*…but I think there’s very much a role with the more junior staff who are learning…they’re the ones who need the explanations more so than the senior people” (P10, Nurse, Senior, Adult)
AI^a^ explanations, cognitive processes, and decision-making (n=13)	
Offset cognitive overload and prompt safety checks (n=7).	“…when there are times of cognitive overload, something that can sort through more information and facilitate a clear direction, that would be much better.” (P9, Nurse, Junior, Pediatric)
Provide deterministic explanations that fit the clinician’s mental model (n=7).	“…So the method two makes a lot more sense because…it fits with my ground truth...” (P14, Doctor, Senior, Neonatal)
Provide plausible explanations that can either reinforce or challenge a clinician’s existing perspective (n=4).	“If it’s a plausible explanation, then obviously I would have to rethink to determine whether that is the right thing.” (P7, Doctor, Senior, Adult)
Avoid ambiguous explanatory factors as they may cause confusion (n=3).	“…the point you provide me with some information that comes out of context, am I better off or not? It is sufficient to make me less certain…” (P1, Doctor, Senior, Adult)
Application of AI CDS^b^ explanations (n=13)	
Prompt patient review and be considered within the broader context of patient-related factors (n=12).	“…there’s so many variables outside of obs that allow us to make decisions.” (P5, Doctor, Junior, Adult)
In the setting of multiple explanations, can prompt the same clinical decision (n=3).	“The fact they don’t agree is not [an issue], I can think of many examples where there are evidence that allowed us to reach the same conclusion...” (P1, Doctor, Senior, Adult)
Trust	
Influencing factors (n=12)	
Clinician age and experience (n=5).	“…the younger the clinicians are, the less they’re frightened of it, maybe the more accepting they are of it…” (P10, Nurse, Senior, Adult)
Accurate predictions, which are trusted more than accurate explanations (n=6).	“…if it triggers that there’s something going wrong, I would probably always take that seriously and so even if the explanation’s not that great, it’s up to us to…rule out what’s going on with the patient…” (P13, Nurse, Senior, Adult)
Conflicts in decision-making stemming from information provided by AI-driven CDS (n=1).	“…these sorts of tools…become kind of bargaining chips between clinical team, because…data that is in black and white…will always be trusted more than someone’s gut feeling or someone’s intuition etc.” (P5, Doctor, Junior, Adult)
Lived clinical experience and clinicians’ understanding of the patient’s situation (n=3).	“…the benefit for these systems is for less experienced people that haven’t got…lived experience clinically…or my senior colleagues because we can’t be at the bedside 24/7.” (P14, Doctor, Senior, Neonatal)
Opinion leaders (n=2).	“…we want the clinicians who have some expertise in managing deteriorating patients to endorse and support the algorithm.” (P7, Doctor, Senior, Adult)
Evidence-based and clinical utility (n=9).	“…if you demonstrated that in the type of hospital I am in, with the type of electronic systems and everything being similar, that the system always was better than all of the clinicians…. I would have to trust it more quickly....” (P2, Doctor, Mid, Adult)
Clinician internal calibration (n=11)	
Repeated exposure, consistent performance, reflection on clinical outcomes, and accuracy of predictions (n=11).	“…having that explainable element and…familiarity with using and trying previous AI or decision support tools successfully and that being helpful in my practice - would influence my views...” (P4, Doctor, Mid, Adult)
Impact of agreement level (n=12)	
Alignment with a clinician’s mental model fosters trust (n=12).	“…what is it about various elements of the patient’s profile that have made that tool perform in such a different way to what I was thinking...” (P4, Doctor, Mid, Adult)
Design recommendation for AI-driven CDS with XAI^c^	
Explanations are actionable and useful (n=10)	
Systems should present actionable data such as physiological trends, rather than standalone routine metrics, in a timely manner (n=10).	“…that’s fairly well understood that the longer somebody stays the more at risk they are for hospital acquired complications.... It doesn’t necessarily value add in that, but the physiological variables definitely.” (P10, Nurse, Senior, Adult)
Systems should demonstrate in-depth predictive ability with detailed analysis of potential reasons for patient deterioration (n=3).	“…rather than just triggering for a temperature or a heart rate…, really putting it up front to say that it would be a sepsis trigger.” (P9, Nurse, Junior, Pediatric)
Patient complexity and contextual factors (n=9)	
Provision of information beyond physiological data, such as the patient’s history and comorbidities (n=9).	“…get the machine learning to pick up that…historical background of pre-admission and intra-admission factors...” (P9, Nurse, Junior, Pediatric)
Other recommendations (n=13)	
Simple, optional, and concise explanations (n=7).	“…the prediction that you had popping up as an overlay above the observations so that people could read them together would be helpful. Along with an explanation as to why, well even do you want the explanation?” (P12, Nurse, Senior, Adult)
The level of certainty and the potential for bias in prediction (n=6).	“…you need shades of confidence…maybe with confidence intervals, not just a score but some idea of how confident the system is.” (P2, Doctor, Mid, Adult)
At-a-glance insights using clear graphics and minimal text (n=5).	“…if it took a lot of time and you had to make a decision about something…, then that would be kind of wasteful or distracting you from a patient.” (P4, Doctor, Mid, Adult)
Color coding to indicate level of deterioration and transparent explanation (n=3).	“…whatever pops up on screen there’s a little bit of explanatory detail and a bit of color coding will be that traffic lights or something else, basically saying this is what the AI model is predicting or directing…and this is why...” (P11, Nurse, Mid, Pediatric)
Information confined to specific intended use cases (n=2).	“So I trust this to give me a list of people that I should see, as a high priority and I can plan my workflow.... I do not trust it to tell me what dose of drug to use and what drugs to use on people.” (P1, Doctor, Senior, Adult)
User-friendly, intuitive, and requiring minimal effort to use (n=1).	“The ones that actually get used clinically are the ones that require the least amount of effort...” (P5, Doctor, Junior, Adult)

a AI: artificial intelligence.

b CDS: clinical decision support.

c XAI: explainable artificial intelligence.

#### Explainability and Actionability

The domain explored when and why explainability is needed in CDS tools. It identified how effective explanations can enhance decision-making by helping clinicians manage cognitive load and focus on key details. It also explored how explanations influence decision-making, particularly when varying levels of disagreement arise between different explanation methods.

Subconstructs explored the context-dependent usefulness of explanations, where the need for explainability varies with clinician experience, task complexity, and decision type. Junior clinicians would benefit more from explanations for understanding AI outputs, especially in unfamiliar or complex situations, while reliance on detailed explanations would decrease in routine tasks or for experienced clinicians who intuitively assess clinical indicators. Junior clinicians may benefit from detailed explanations because they are still developing their clinical reasoning and may rely on AI outputs as a learning tool. In contrast, senior clinicians, drawing on extensive experience, may use AI mainly as a confirmatory aid, focusing on explanations primarily when outputs diverge from their expectations or offer novel insights. As AI tools may fail to capture the multifactorial nature of patient conditions and physiology, their role should be to provide direction on which physiological parameters to investigate further, with final decision-making remaining a human responsibility.

#### Trust

The trust domain examined factors influencing trust in AI-driven CDS, including clinicians’ age, experience, intuition alignment, and the tool’s predictive accuracy. It further highlighted how trust builds gradually through reliable performance and repeated use, enabling integration with clinical decisions. Specifically, confidence would grow as users understand the evidence behind the algorithm and how it was developed. Validation of the XAI tools by multiple studies and investigators, particularly if in similar settings, would similarly foster trust. One of the most important determinants of trust was alignment with clinical instinct. A system that consistently produced reliable outcomes containing few discrepancies with expert clinical judgment would increase trust, while contradictions would undermine trust, particularly when there is a disagreement with the tool’s predictions.

#### Design Recommendations for AI-Driven CDS With XAI

This domain outlines key design considerations for AI-driven CDS tools from the participants, focusing on when and how explanations are presented. It highlights delivering actionable, contextualized information through clear, concise explanations that focus on physiological trends and patient history. While centered on XAI-supported CDS, the findings also underscore the importance of communicating prediction certainty and potential biases to enhance decision-making without overwhelming users.

This means that insights must be actionable, focusing on physiological trends rather than routine metrics, and offering timely and relevant data. A detailed analysis of potential deterioration causes would be preferred over just alerts, with the potential to suggest actions such as specific tests to order. The ability to integrate additional variables could better predict outcomes, such as mental state deterioration, past clinical history, environmental factors, laboratory values, historical data, and, in pediatric settings, parental concerns.

From a user interface perspective, AI tools with concise and customizable presentation options would allow for variations in experience and complexity. Clear, fact-based explanations using bullet points or likelihood percentages were optimal for busy critical care settings, with additional guidance available if required. The provision of weighted graphs, CIs, graded risk indicators, and visual cues such as traffic-light systems allowed clinicians to assess and mitigate risk.

## Discussion

### Principal Findings

This study explored how explainability influences clinicians’ trust, interpretation, and use of AI-driven CDS tools in the context of Australian critical care settings, with particular attention to how clinicians respond when different explainability methods produce different explanations for the same AI prediction. While trust and explainability have been explored in prior research [1,27,29,30,32,47-50], this study contributes novel insights by addressing the underexamined issue of discrepancies between explanation methods, a phenomenon increasingly recognized in technical XAI research [37,42-46] but not studied from a human-centered or clinical perspective. Our findings highlighted a broad range of factors that influence clinicians’ use of and trust in AI-driven CDS tools, including how explanations are perceived, applied, and deemed useful, all of which directly informed key design and implementation considerations for XAI-driven CDS systems. The study also offers a novel contribution to the understanding of how explainability influences the determinants for establishing initial trust and overtime trust in AI-driven tools. Specifically, we delineate the positioning of explainability in supporting both perceived usefulness (for initial trust) and learned usefulness (for overtime trust) [58].

The study investigated the perceptions of Australian clinical experts on using explanations as part of their clinical decision-making and how explanations impact trust in XAI-driven CDS. The findings directly address RQ1 by answering how explainability influences clinicians’ use, trust, and perceived usefulness of AI-driven CDS tools. The results showed that explainability plays a dynamic and context-dependent role in shaping clinicians’ interactions with these systems. Consistent with the literature [29,59], the clinical experts in our study agreed that explanations are a valuable feature that enhances trust and the usefulness of AI-driven CDS tools. However, the necessity and value of explanations vary depending on the context and the clinician’s experience. The importance of context-based explanations was similarly highlighted in the findings of Barda et al [60]. For more fundamental decision-making (such as bed allocation, ordering routine blood tests, and administering pain relief), explanations are often unnecessary and can be viewed as an optional resource. Additionally, experienced clinicians may rely more on their own clinical intuition and use explanations less frequently, while less experienced clinicians may prefer more detailed explanations to guide their decision-making. A similar finding was reported by Panigutti et al [49], where the explanation interface was found to be valuable in helping novices avoid mistakes and supporting collaborative decision-making tasks.

Drawing upon the integrative trust model proposed by Cabiddue et al [58], which identified perceived usefulness as a crucial determinant of initial trust in AI-driven tools, our findings reveal a stronger need for explainability during this nascent stage. Explainability is shown to be particularly instrumental in supporting perceived usefulness, especially when users are developing familiarity with the tool. Furthermore, the value of explainability appears to be moderated by user expertise. Novice users, who lack extensive clinical experience, may find explanations more useful in establishing initial trust compared to their expert counterparts. Conversely, the model highlighted learned usefulness as the key determinant for establishing overtime trust. A key finding of our study is that the importance of explainability diminishes overtime as users transition from perceived usefulness to learned usefulness. For expert users, in particular, the significance of explainability decreases notably once they have established confidence in the tool’s predictions and rely more heavily on their clinical judgment. Once this level of trust is solidified, the contribution of explainability to learned usefulness becomes less pronounced.

The study further investigated whether partial or complete discrepancies between different XAI methods affect the trust and perceived usefulness of the tool. Participants viewed AI recommendations as an adjunct in decision-making and were not concerned by discrepancies in explanations, prioritizing their own patient assessments. They stressed the need for accurate AI predictions to maintain trust and avoid alarm fatigue. Explanations were found most helpful when they aligned with clinicians’ mental models and the patient’s holistic condition. However, participants emphasized that the initial prognostic or diagnostic model predictions must be accurate, as too many false positives would erode trust in the system and lead to alarm fatigue. Alignment with clinicians’ mental models is crucial for adopting AI-driven tools [1], as explanations that match these models foster trust, while large discrepancies can cause skepticism. Our study found that discrepancies in explanations enabled reflection through skepticism [59], a fundamental concept in human-centered XAI that encourages critical thinking over passive acceptance when evaluating explanations and building trust in the system [61]. In addition, clinicians often face complex decisions where different approaches yield the same outcome; explanations aligning with their thinking, despite discrepancies in key factors, did not significantly affect trust. However, repeated poor performance in predictions and explanations can lead to loss of trust, consistent with prior findings linking high AI performance to greater acceptance [62,63]. While previous studies have explored XAI’s usefulness [64-67] and compared AI’s prediction and clinical judgments [19,67,68], few have focused on how explanation discrepancies impact clinician decision-making. Addressing RQ2, our findings show that clinicians may tolerate some level of discrepancies between explanation methods as long as the AI prediction is accurate and the explanations remain clinically meaningful. Rather than dismissing the system, they respond evaluatively, using discrepancies to scrutinize the output, while maintaining trust when the system’s outputs are accurate, actionable, and aligned with their clinical reasoning.

Finally, the study identified several design recommendations for successful use of XAI systems in clinical settings. Explanations should focus on actionable data, emphasizing physiological trends (eg, respiratory rate and blood pressure) over routine metrics such as length of stay. Instead of a single metric, the trend over a specific period of time should be shown to assist in making specific clinical decisions. Providing actionable recommendations, such as potential diagnostic triggers, was also suggested by some participants, a finding that was also echoed in related studies [60,69]. Alongside the explanation when a prediction is generated, AI-driven systems should incorporate contextual information on the patient’s clinical history and clinical complexity. This is consistent with the literature that explored factors influencing medical experts’ use of AI-driven CDS systems [1,70,71]. Multiple studies identified that a lack of a holistic perspective of the patient in the CDS recommendations provided hindered the system’s adoption in the health care settings [50,70]. In adult settings, information on comorbidities and previous admissions provides valuable insights, while in pediatric settings, incorporating concerns from both parents and clinicians offers a more holistic view of the patient’s condition. Some additional contexts that could be included are the underlying causes of physiological changes, including medication effects, movements, and so on. Providing contextual information along with explanations aligns with the mental model of collaborative practice, enabling colleagues to deliberate and discuss the broader context of a patient’s condition to make informed clinical decisions [19].

Explanations need to be simple, concise, and presented in an optional format to avoid overwhelming clinicians. Some ways of visualization include using bullet points, likelihood percentages, and summaries to communicate essential information effectively. The level of detail should be customizable, enabling clinicians of different expertise to adjust the information shown according to their preferences. Ensuring that explanations are digestible would allow clinicians to focus on critical insights without sifting through lengthy text, with additional background and secondary details accessible on demand [72]. Aligned with our findings to prioritize accurate predictions, CDS systems should clearly convey prediction certainty and explanations, along with disclaimers about potential biases. This can be achieved by including CIs or graded risk indicators, alongside visual aids such as weighting graphs on both the predictions and corresponding explanations. Displaying AI confidence scores has been found to enhance trust calibration, leading people to trust the AI more when it shows higher confidence [27,73]. Predictions and their accompanying explanations should be presented within an optimal timeframe to ensure they are actionable, as alerts that come too early or too late can heighten clinician anxiety. The appropriate timing will vary depending on the hospital setting, patient cohort, and available resources, making it crucial to determine this in consultation with expert clinicians. Together, these design considerations demonstrate how explainability, when thoughtfully implemented, underpins trustworthy, context-aware, and user-centered integration of AI-driven CDS tools in clinical settings.

Our study strengthens the human-centered XAI literature by providing evidence from the Australian health care context, addressing a gap in prior research that has predominantly been conducted internationally [1,29,50,59,60,64,65,69,71,74]. We find that key insights from the state-of-the-art literature—such as the value of actionable, context-based explanations, and the variation in reliance on AI by clinician experience—are also relevant in Australian settings. Additionally, the analysis reveals new insights into clinicians’ views and tolerance for discrepancies between explanations. Finally, our results highlight local nuances, including workflow considerations and concerns about overreliance in high-pressure environments, offering practical guidance for the design and deployment of AI-driven CDS tools with XAI in Australia.

### Limitations and Future Work

One limitation of our study is the composition of the participant cohort. ICU settings are among the most critical in health care, where timely and precise decision-making is vital. This focus motivated our approach and guided our selection of clinical experts from this environment. Nonetheless, many of our findings, particularly those related to clinician trust, explanation usefulness, and balancing guidance with overreliance, may also apply to other clinical settings such as emergency departments or surgical units. However, it is important to note that differences in workflow, patient acuity, and team structures could influence how AI-driven CDS tools are used. Though our findings may apply to other domains with certain domain adaptation, further research is needed to assess the generalizability of our results beyond the ICU. Future studies could examine perspectives from these additional settings and incorporate participants from other countries outside Australia to provide a more comprehensive understanding. Due to the study’s qualitative nature, the findings should be interpreted with caution when considering generalizability. It is important to acknowledge that the scenarios used in this study were based on a desktop prototype, which may have constrained participants’ ability to fully consider the scenarios within the context of real-world decision-making environments. Future research could explore the use of more interactive and immersive prototypes, such as those integrated into clinical workflows or simulated environments, to better capture the nuances of decision-making in practice. These future studies could include large-scale usability studies to assess the impact of these actionable insights and further support our qualitative conclusions. Further research should explore scenarios in which the CDS provides plausible yet conflicting predictions relative to clinicians’ expected reasoning. Examining such cases would offer deeper insights into how explanations influence trust calibration and decision-making under conditions of disagreement. Future research could also focus on validating the design recommendations for AI-driven CDS tools, ensuring they are adaptable to various clinical scenarios and effectively address trust, safety, and usability concerns, thereby supporting widespread and ethical adoption in health care. This study contributed to the development of a checklist for XAI tools [75]. Evaluating explanations in development and trial phases using the checklist could help enhance trust, reduce risks, foster AI adoption, and determine the true clinical potential of applied XAI.

### Conclusions

This study identified recommendations for developing trustworthy, user-centric XAI-supported CDS tools. Three key domains were identified, emphasizing the importance of trust and explainability in gaining clinician acceptance of AI tools. It also highlighted that discrepancies between different explanation techniques are not inherently problematic, provided the explanations are consistent with clinicians’ mental model. While developing and deploying algorithms is relatively low-cost, the greater challenge lies in integrating them into clinical workflows, requiring acceptance by health care professionals. By examining user needs, our findings support the development of clinician-informed, actionable insights throughout the AI tool lifecycle. This approach can enhance trust, mitigate risks, and promote sustainable health care by aligning AI systems with clinical workflows and improving long-term patient outcomes through informed and effective use.

## Supplementary material

10.2196/81460 Multimedia Appendix 1 Semistructured interview guide.

10.2196/81460 Multimedia Appendix 2 Detailed scenarios presented to participants in the semistructured interview.

10.2196/81460 Multimedia Appendix 3 Additional qualitative findings that did not directly address the research question.

10.2196/81460 Checklist 1 COREQ (Consolidated Criteria for Reporting Qualitative Research) checklist.

## Abbreviations

AI: artificial intelligence
CDS: clinical decision support
COREQ: Consolidated Criteria for Reporting Qualitative Research
CSIRO: Commonwealth Scientific and Industrial Research Organisation
ICU: intensive care unit
REDCap: Research Electronic Data Capture
RQ: research question
XAI: explainable artificial intelligence
