# Supplementary material for: The Role of Explanations in AI-Generated Alerts: Qualitative Study of Clinical Views on Explainable AI in Predictive Tools
Source: JMIR Hum Factors. 2026 May 1;13:e81460. doi: 10.2196/81460 (PMC13134825; doi:10.2196/81460)
Supplement: Multimedia Appendix 1 [file humanfactors-v13-e81460-s001.docx]

Semistructured interview guide

| Introduction | We have developed a machine learning model that can detect patient deterioration. In its’ original form, the model tells the clinician the likelihood that a patient will trigger a vital sign alert within the next four hours, but it does not tell them why or what parameter triggered the alert. The team are developing an explainability module whereby the clinicians will know what vital sign or parameter likely triggered the alarm, providing more specific support for clinical decision-making.  We will ask you some questions about your views on clinical decision support tools that use predictive algorithms and the role of the explainability module. The session will be audio-recorded for transcription purposes. Please ensure you do not share any confidential information. Throughout the session please do not provide any identifying information about anyone else like other staff or patients.  Below are a few definitions to help you understand what we are discussing.  Clinical Decision Support Systems (CDSS) are “computer systems designed to impact clinician decision making about individual patients at the point in time that these decisions are made”.  Predictive algorithms are derived “using computations to map data on patient factors to target outcomes, such as death or cardiac arrest, rather than relying on a human expertise and clinical judgement. Such predictive algorithms are developed using real, historical patient data sets”.  Explainability: means “the ability of AI to justify its outcomes and assist clinicians in rationalising the model prediction”. It is defined as, “a set of measurable, quantifiable, and transferable attributes associated with an AI system targeted for clinicians to calibrate model trust”.  Trust in these systems is defined as, “the extent to which [you are] confident in, and willing to act on the basis of, the recommendations, actions, and decisions of these systems”. |
| --- | --- |
| **Broad, opening questions** | - **Please state your role and how long have you been in this role** - **When you hear the phrase “AI-driven prediction algorithm for Clinical Decision Support Systems what do you think of?** - Do you have any experience in using Clinical Decision Support Systems? (Only use this if the answer doesn’t come during the previous response) - If so, can you tell me about your experience? (PROBE What do you think of it? Is it helpful?) - **How useful do you think Clinical Decision Support Systems are for assisting you with your job/practice?** - **When I say explanation of the prediction made by CDSS what do you think of?** |
| **Trust & Explainability** | - **What are the factors that are most predictive of a positive patient outcome such as, going back to the ward?**    - What are the factors for negative outcomes such as further deterioration? - **If a piece of software gave you some information that could potentially influence your clinical decision, how would you feel about that?**    - What would make you trust/doubt it? - **How would you compare the tool’s decision with your own expert clinical knowledge and judgement?**   (Two types of PROBE How would you make a decision when the algorithm’s prediction aligns with your clinical knowledge? How would you make decision when it doesn’t align?)  (Follow up: how would you use it? When is it not needed)) |
| **Discordance** | We have previously talked about tool’s prediction aligning or not aligning with your judgement. Now we’ll focus on the explanations of the predictions generated.   - **If the software gives you an explanation for its output, that you agree with, how would this make you feel?**   1. **What about if it partially aligns?**   2. **What about if it doesn’t align?** - (**start of scenario**) Let’s assume the system at the hospital you are working is using an algorithm that predicts the activation of an early warning system (EWS). A patient in the system is showing red flag indicating a high risk of patient deterioration. As a clinician, you know this one has the potential to deteriorate with respiratory failure because of the vital signs trends (low respiratory rate, and low systolic blood pressure) and patient conditions are characteristic for respiratory failure. Let’s assume at the beck-end there are two different algorithms running.   **Use case 1**: explanations of both algorithms partially agree with your clinical knowledge but are not the same (show the use case outlined below)  **Use case 2**: explanations of both algorithms are different from your clinical knowledge. (show the use case outlined below)  After each use cases ask the following questions   - 1. **How does it affect your decision-making/judgement?**   2. **How would it impact your trust in the system?** - Follow up: Would you like to mention any such example use cases based on your personal experience? - **What components would you like to see in the explanation of a prediction generated by AI? (You may need to prompt them here with examples)** - What is your view on explanations as a part of AI-supported CDS systems, is it need and why? Tell me a scenario when the explanation of a prediction is needed? |
| **General** | - Any other additional comments based on today’s discussion? - Can we meet with you again? |
