# Supplementary material for: The Role of Explanations in AI-Generated Alerts: Qualitative Study of Clinical Views on Explainable AI in Predictive Tools
Source: JMIR Hum Factors. 2026 May 1;13:e81460. doi: 10.2196/81460 (PMC13134825; doi:10.2196/81460)
Supplement: Multimedia Appendix 2 [file humanfactors-v13-e81460-s002.pdf]

Scenario 1

A 53-year-old man was admitted for a pressure ulcer of the buttock and has been on ward 9 for 8 days.

Procedures

Proc_Desc	Surg_Proc	Proc_Snomed	Proc_Source_Ident	Terminology_Axis	Surg_Specialty	Anesth_Type	Proc_Start_DT_TM	Proc_End_DT_TM
Debridement of skin and subcutaneous tissue	Debridement of skin and subcutaneous tissue	SNOMED!31064000	51988013	Procedure	Plastics SN	General	22/02/2016 16:09	22/02/2016 16:39
Laparoscopy	Laparoscopy	SNOMED!73632009	122272015	Procedure	Colorectal SN	General	22/02/2016 18:06	22/02/2016 18:37
Laparotomy	Laparotomy	SNOMED!86481000	143418010	Procedure	Colorectal SN	General	22/02/2016 18:38	22/02/2016 22:28
Lysis of adhesions	Lysis of adhesions	SNOMED!39270003	65851019	Procedure	Colorectal SN	General	22/02/2016 19:39	22/02/2016 22:28
Permanent colostomy	Permanent colostomy	SNOMED!4044002	7964016	Procedure	Colorectal SN	General	22/02/2016 16:09	22/02/2016 22:28

Proc_Text	Sched_Surg_Proc	Sched_Primary_Ind	Sched_Surg_Specialty	Sched_Anesth_Type	Sched_Seq_Num	Updt_DT_TM
WASHOUT + DEBRIDE OF SACRAL PRESSURE AREA + EUA + DEFUNCTIONING COLOSTOMY	Debridement of skin and subcutaneous tissue	1	Plastics SN	General	1	22/02/2016 9:18
WASHOUT + DEBRIDE OF SACRAL PRESSURE AREA + EUA +DEFUNCTIONING COLOSTOMY	Laparoscopy	0			2	22/02/2016 9:18
WASHOUT + DEBRIDE OF SACRAL PRESSURE AREA + EUA +DEFUNCTIONING COLOSTOMY	Laparotomy	0			3	22/02/2016 13:03
	Lysis of adhesions	0			4	22/02/2016 13:03
	Permanent colostomy	0			5	22/02/2016 13:03

OFFICIAL

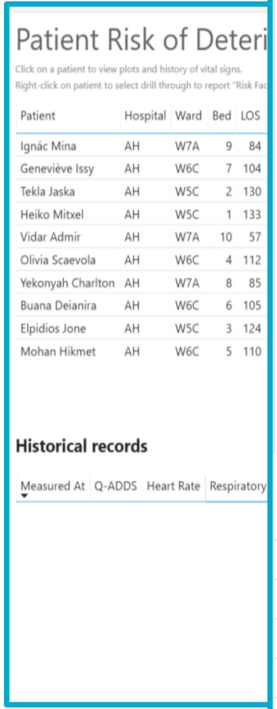

Patient Risk of Deterioration

Click on a patient to view plots and history of vital signs.
Right-click on patient to select drill through to report "Risk Factors".

Patient	Hospital	Ward	Bed	LOS	QADDS	HR	RR	SBP	DBP	Temp	DRS
Ignác Mina	AH	W7A	9	84	[illegible]	[illegible]	[illegible]	[illegible]	[illegible]	[illegible]	[illegible]
Geneviève Issy	AH	W6C	7	104	[illegible]	[illegible]	[illegible]	[illegible]	[illegible]	[illegible]	[illegible]
Tekla Jaska	AH	W5C	2	130	[illegible]	[illegible]	[illegible]	[illegible]	[illegible]	[illegible]	[illegible]
Heiko Mitxel	AH	W5C	1	133	[illegible]	[illegible]	[illegible]	[illegible]	[illegible]	[illegible]	[illegible]
Vidar Admir	AH	W7A	10	57	[illegible]	[illegible]	[illegible]	[illegible]	[illegible]	[illegible]	[illegible]
Olivia Scaevola	AH	W6C	4	112	[illegible]	[illegible]	[illegible]	[illegible]	[illegible]	[illegible]	[illegible]
Yekonyah Charlton	AH	W7A	8	85	[illegible]	[illegible]	[illegible]	[illegible]	[illegible]	[illegible]	[illegible]
Buana Deianira	AH	W6C	6	105	5	105	28	135	75	38.50	60
Elpidios Jone	AH	W5C	3	124	3	105	20	142	76	37.10	36
Mohan Hikmet	AH	W6C	5	110	2	109	13	105	70	36.80	24

Ignac Mina	W7A
Patient	Ward
52.9	9
Patient ID	Bed
AH	8.4 d
Hospital	LOS
23/02/2016 0:31	
Latest Measure	

Historical records

Date and Time	SBP	DBP	Temp	Heart Rate	Resp Rate	SpO2	AP
22/02/2016 23:19	105	54	36.4	78	14	100	71
22/02/2016 23:24	98	56	36.4	75	16	100	70
22/02/2016 23:39	110	59	36.4	68	16	100	76
22/02/2016 23:53	119	89	36.4	72	15	100	99
23/02/2016 0:13	101	58	36.4	70	14	100	72
23/02/2016 0:16	102	55	36.4	69	16	100	71
23/02/2016 0:31	99	58	35.9	67	14	100	72

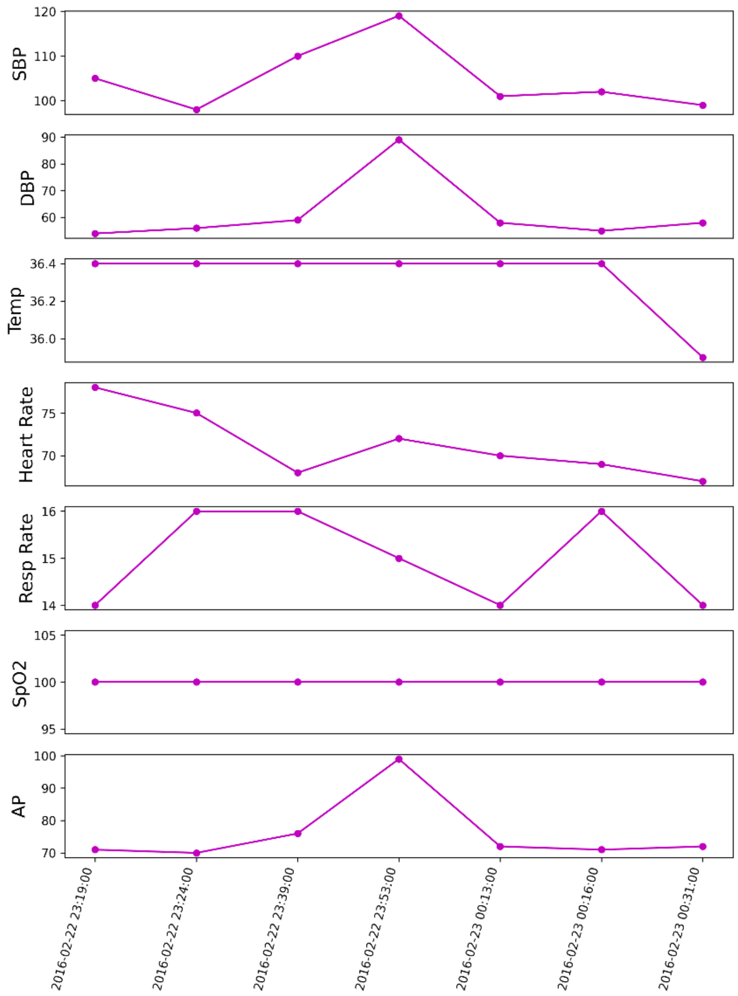

Dashboard Mock-up – Risk Profile

Predicted patient deterioration within next 8 hours with highest risk – Risk 1

Patient Deterioration Risk Factors

Ignac Mina
Patient

67
Patient ID

AH
Hospital

W7A
Ward

9
Bed

8.4 days
LOS

23/02/2016 0:31
Latest Measurement

Ground truth

EWS: Triggered
DT: 23/02/2016 8:15am
EWS trigger: BP

Dashboard Mock-up – Risk Profile

Predicted patient deterioration within next 8 hours with highest risk – Risk 1

Patient Deterioration Risk Factors

Ignac Mina
Patient

Risk 1

67
Patient ID

AH
Hospital

W7A
Ward

9
Bed

8.4 days
LOS

23/02/2016 0:31
Latest Measurement

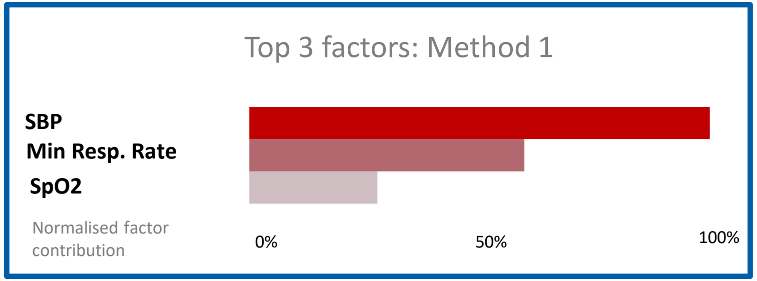

OFFICIAL

Dashboard Mock-up – Risk Profile

Predicted patient deterioration within next 8 hours with highest risk – Risk 1

Patient Deterioration Risk Factors

Ignac Mina
Patient

Risk
1

67
Patient ID

AH
Hospital

W7A
Ward

9
Bed

8.4 days
LOS

23/02/2016 0:31
Latest Measurement

Top 3 factors: Method 2

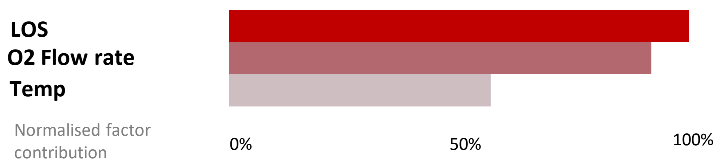

OFFICIAL

OFFICIAL

Dashboard Mock-up – Risk Profile

Predicted patient deterioration within next 8 hours with highest risk – Risk 1

Ignac Mina
Patient

67
Patient ID

AH
Hospital

W7A
Ward

9
Bed

8.4 days
LOS

23/02/2016 0:31
Latest Measurement

Risk 1

Patient Deterioration Risk Factors

Top 3 factors: Method 1

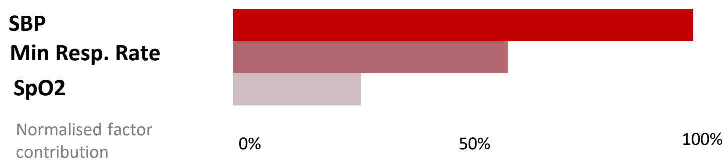

Top 3 factors: Method 2

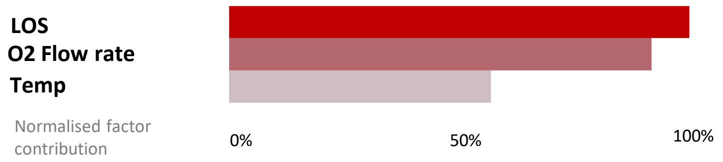

Ground truth

EWS: Triggered
DT: 23/02/2016 8:15am
EWS trigger: BP

OFFICIAL

Scenario 2

A 67-year-old man was admitted for limb/joint pain and has been on ward 9 for 8 days.

OFFICIAL

Procedures

Proc_Desc	Surg_Proc	Proc_Snomed	Proc_Source_Ident	Terminology_Axis	Surg_Specialty	Anesth_Type	Proc_Start_DT_TM	Proc_End_DT_TM
Open reduction of fracture of tibial plateau with internal fixation	Open reduction of fracture of tibial plateau with internal fixation	SNOMED!2761000032104	5.51E+12	Procedure	Orthopaedic SN	General	18/10/2017 15:22	18/10/2017 17:30
Bladder catheterisation	Bladder catheterisation	SNOMED!410024004	2.48E+09	Procedure	Orthopaedic SN	General	18/10/2017 13:52	18/10/2017 13:54
Open reduction of fracture of foot with internal fixation	Open reduction of fracture of foot with internal fixation	SNOMED!359934004	4.76E+08	Procedure	Orthopaedic SN	General	18/10/2017 14:08	18/10/2017 15:22

Proc_Text	Sched_Surg_Proc	Sched_Primary_Ind	Sched_Surg_Specialty	Sched_Anesth_Type	Sched_Seq_Num	Updt_DT_TM
(P1) RIGHT CALCANEAL FRACTURE ORIF +LEFT TIBIAL PLAFONDÂ # ORIF	Open reduction of fracture of tibial plateau with internal fixation	1	Orthopaedic SN	General	1	46:25.0
(P1) RIGHT CALCANEAL FRACTURE ORIF +LEFT TIBIAL PLAFONDÂ # ORIF	Bladder catheterisation	0			2	47:11.0
(P1) RIGHT CALCANEAL FRACTURE ORIF +LEFT TIBIAL PLAFONDÂ # ORIF	Open reduction of fracture of foot with internal fixation	0			3	47:08.0
Proc_Text	Sched_Surg_Proc	Sched_Primary_Ind	Sched_Surg_Specialty	Sched_Anesth_Type	Sched_Seq_Num	Updt_DT_TM
(P1) RIGHT CALCANEAL FRACTURE ORIF +LEFT TIBIAL PLAFONDÂ # ORIF	Open reduction of fracture of tibial plateau with internal fixation	1	Orthopaedic SN	General	1	46:25.0

OFFICIAL

OFFICIAL

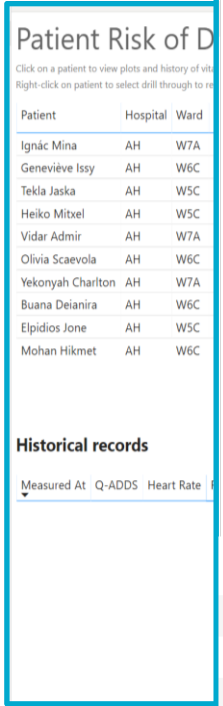

Patient Risk of Deterioration

Click on a patient to view plots and history of vital signs.
Right-click on patient to select drill through to report "Risk Factors".

Patient	Hospital	Ward	Bed	LOS	QADDS	HR	RR	SBP	DBP	Temp	DRS
Ignác Mina	AH	W7A	9	84	[illegible]	[illegible]	[illegible]	[illegible]	[illegible]	[illegible]	[illegible]
Geneviève Issy	AH	W6C	7	104	[illegible]	[illegible]	[illegible]	[illegible]	[illegible]	[illegible]	[illegible]
Tekla Jaska	AH	W5C	2	130	[illegible]	[illegible]	[illegible]	[illegible]	[illegible]	[illegible]	[illegible]
Heiko Mitxel	AH	W5C	1	133	[illegible]	[illegible]	[illegible]	[illegible]	[illegible]	[illegible]	[illegible]
Vidar Admir	AH	W7A	10	57	[illegible]	[illegible]	[illegible]	[illegible]	[illegible]	[illegible]	[illegible]
Olivia Scaevola	AH	W6C	4	112	[illegible]	[illegible]	[illegible]	[illegible]	[illegible]	[illegible]	[illegible]
Yekonyah Charlton	AH	W7A	8	85	[illegible]	[illegible]	[illegible]	[illegible]	[illegible]	[illegible]	[illegible]
Buana Deianira	AH	W6C	6	105	5	105	28	135	75	38.50	60
Elpidios Jone	AH	W5C	3	124	3	105	20	142	76	37.10	36
Mohan Hikmet	AH	W6C	5	110	2	109	13	105	70	36.80	24

Tekla Jaska	W7A
Patient	Ward
67.5	9
Patient ID	Bed
AH	8.17 d
Hospital	LOS
18/10/2017 17:48	
Latest Measure	

Historical records

Date and Time	SBP	DBP	Temp	Heart Rate	Resp Rate	SpO2	AP
2017-10-17 11:55:23	122	82	36.4	93	16	95	96
2017-10-17 15:21:00	112	69	36.7	74	16	95	83
2017-10-17 19:10:00	128	78	36.7	88	17	97	95
2017-10-17 22:43:12	117	56	36.7	87	16	97	76
2017-10-18 08:36:00	117	56	36.7	87	17	97	76
2017-10-18 08:36:45	117	75	36.3	84	17	96	89
2017-10-18 17:48:00	125	87	36.4	63	11	99	100

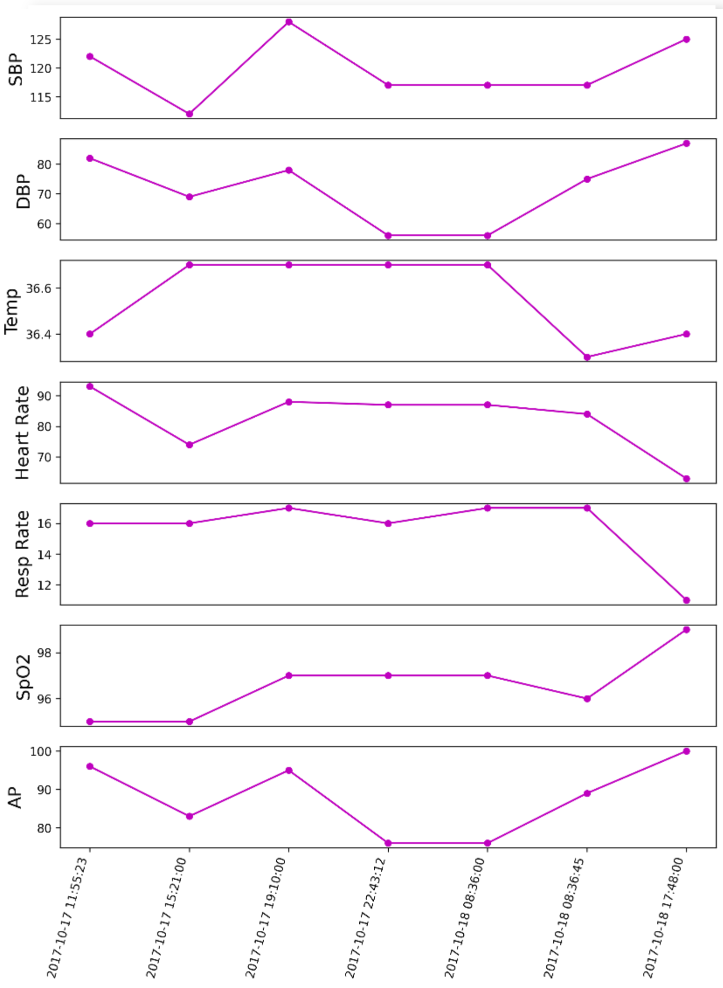

OFFICIAL

Dashboard Mock-up – Risk Profile

Predicted patient deterioration within next 8 hours with highest risk – Risk 1

Patient Deterioration Risk Factors

Tekla Jaska
Patient

67
Patient ID

AH
Hospital

W7A
Ward

9
Bed

8.17 days
LOS

18/10/2017 17:48
Latest Measurement

Ground truth

EWS: Triggered
DT: 18/10/2017 18:28
EWS trigger: Resp Rate

OFFICIAL

Dashboard Mock-up – Risk Profile

Predicted patient deterioration within next 8 hours with highest risk – Risk 1

Patient Deterioration Risk Factors

Tekla Jaska
Patient

Risk
1

67
Patient ID

AH
Hospital

W7A
Ward

9
Bed

8.17 days
LOS

18/10/2017 17:48
Latest Measurement

Top 3 factors: Method 1

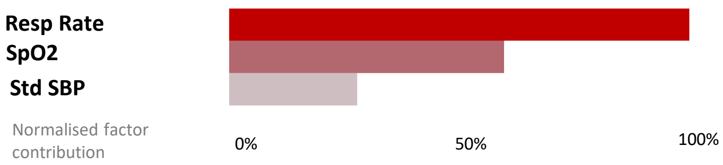

OFFICIAL

OFFICIAL

Dashboard Mock-up – Risk Profile

Predicted patient deterioration within next 8 hours with highest risk – Risk 1

Patient Deterioration Risk Factors

Tekla Jaska
Patient

Risk
1

67
Patient ID

AH
Hospital

W7A
Ward

9
Bed

8.17 days
LOS

18/10/2017 17:48
Latest Measurement

Top 3 factors: Method 1

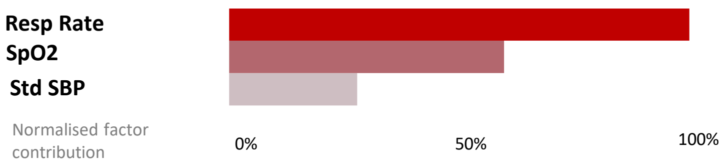

Top 3 factors: Method 2

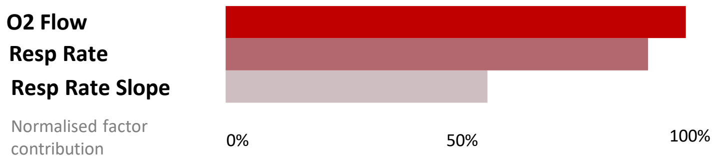

OFFICIAL

Dashboard Mock-up – Risk Profile

Predicted patient deterioration within next 8 hours with highest risk – Risk 1

Patient Deterioration Risk Factors

Tekla Jaska
Patient

67
Patient ID

AH
Hospital

W7A
Ward

9
Bed

8.17 days
LOS

18/10/2017 17:48
Latest Measurement

Risk 1

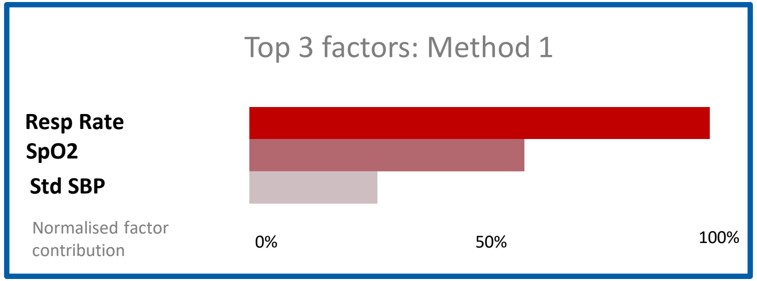

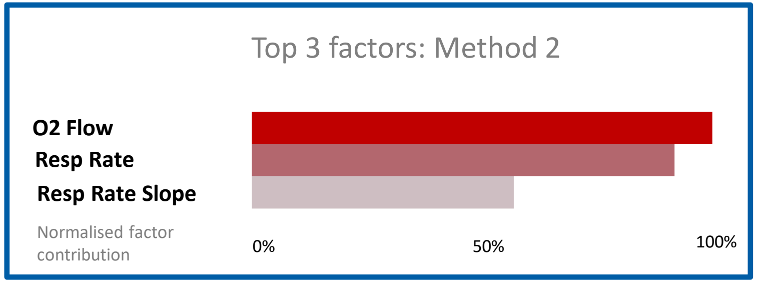

Ground truth

EWS: Triggered
DT: 18/10/2017 18:28
EWS trigger: Resp Rate
