# Supplementary material for: The Role of Explanations in AI-Generated Alerts: Qualitative Study of Clinical Views on Explainable AI in Predictive Tools
Source: JMIR Hum Factors. 2026 May 1;13:e81460. doi: 10.2196/81460 (PMC13134825; doi:10.2196/81460)
Supplement: Multimedia Appendix 3 [file humanfactors-v13-e81460-s003.docx]

### Additional Findings

#### Use of AI-Powered Clinical Decision Support Systems

This domain explored where and for whom these tools are most beneficial. It highlighted how AI can support clinical judgement by helping clinicians manage complex data, leading to enhanced patient safety and care accuracy. AI is considered useful for patient triage or monitoring, complex data, and busy environments. AI was perceived as enhancing patient safety by prompting clinicians to reassess patients and identify early signs of deterioration.

Sub-constructs explored the role of AI tools in early intervention, such as identifying changes in heart or respiratory rates that may indicate sepsis, serving as an additional monitoring layer, and flagging issues overlooked due to cognitive overload or busy environments. This role comes to the fore in critical care, where large-scale complex data and interactions from multiple sources require prioritisation, triaging, and resource allocation. AI could potentially relieve pressure on junior staff, who may lack the experience required to make critical decisions, by highlighting pertinent factors. In some cases, such as stable patients with clear management plans or experienced clinicians, the CDS is seen as a supplementary tool. Here XAI alerts may augment clinical decision-making, prompting revision of patient data and clinical assessment. This supplementary role also supports collaborative assessments among healthcare professionals.

#### Clinicians Concerns and Limitations

This domain reflected the experts' concerns about AI-driven decision support, highlighting risks of over-automation and potential over-reliance by less experienced clinicians. Issues such as alert fatigue, inaccurate or context-lacking information, and deterministic AI responses were sometimes seen as barriers to effective decision-making.

While automation has advantages in streamlining workflows and improving patient outcomes, there is concern that it might undermine critical thinking through an over-reliance on technology without fully understanding the underlying processes or steering clinicians in the wrong direction. Conversely, frequent, irrelevant, or incorrect alerts could lead to disregard of the technology, as these can hinder clinician decision-making. There was also concern that AI may not be ready for complex cases requiring human judgement, with its rigid nature unable to accommodate unmodifiable factors, or those requiring human judgement and flexibility. Finally, there was concern over the anxiety generated by the prediction of events that may not occur.

#### Implementation Considerations

This domain highlights the essential elements for the successful adoption of AI-driven tools in healthcare settings. It highlighted the need for robust research and development (R&D), strong governance, comprehensive training, and mentorship to ensure clinicians understand and appropriately use these tools without compromising critical thinking.

Primarily, implementation should be preceded by robust research, comprising validated evidence, peer-reviewed studies, and robust theoretical frameworks. Studies should be conducted with relevant cohorts, statistically significant samples, transparent error reporting, and benchmarking against best practices to ensure safety and efficacy. AI tool development should have clear regulatory oversight, such as TGA approval and clinical trial adherence, and align with existing guidelines. Once procured, education and training, with mentorship from experienced clinicians, were seen as key to effective adoption, ensuring clinicians understand the tools’ principles rather than relying solely on automation. Integration of the AI-driven CDS would require change management practices facilitated by strong stakeholder engagement.

### Synthesis of Results


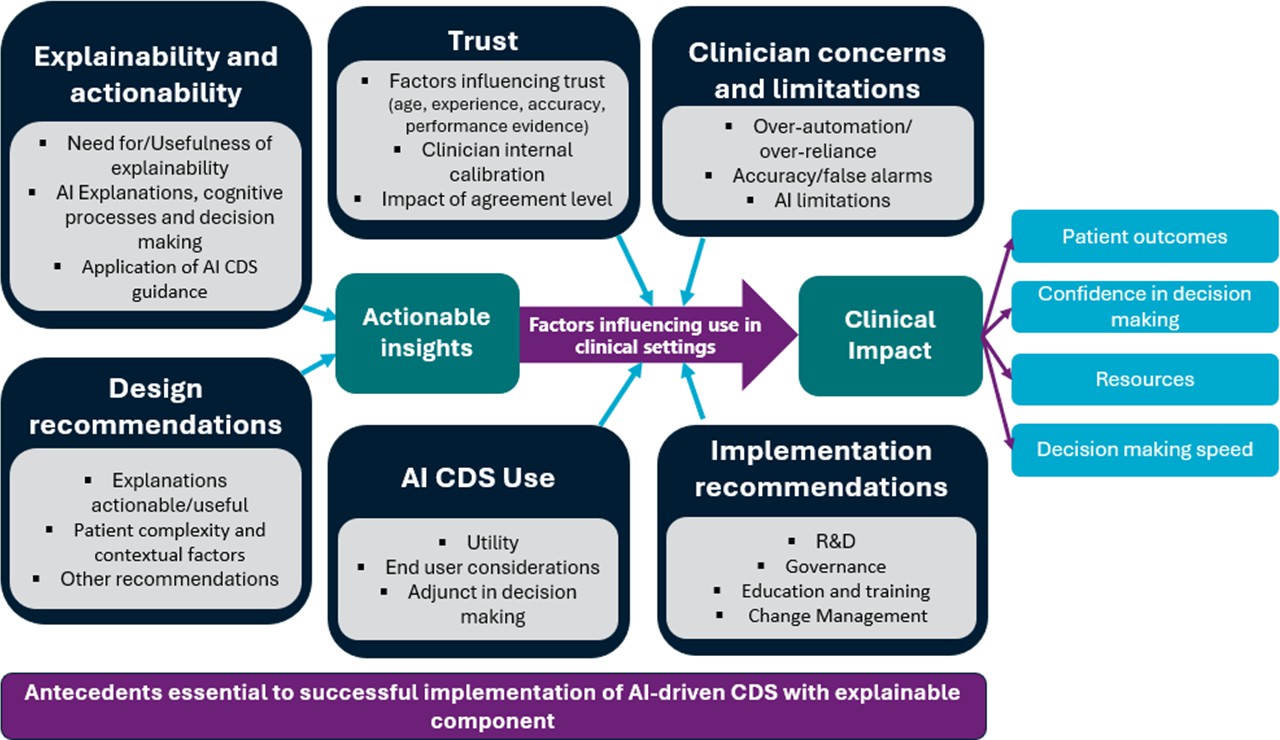
Together, the six domains guide successful development and implementation of AI CDS systems with XAI in clinical settings to influence decision making, resource allocation, and ultimately, improve patient outcomes.

Figure S1: Diagram illustrating the operationalisation of the themes for identifying key considerations in implementing XAI-driven clinical decision support (CDS) systems.

Table S1. Additional domains, constructs, and subconstructs were identified in the qualitative analysis.

| **Domain** | **Construct (n = Number of participants)** | **Sub-Construct (n = Number of participants)** | **Exemplar quotes** |
| --- | --- | --- | --- |
| **Use of AI powered decision support systems** | **Utility** **(n=13)** | Provides assistance with navigation of large-scale complex data to focus on critical information. (n=12) | *“…pulling those altogether, making it easy for humans to make the right decisions …”* (P6, Nurse, Senior, Paediatric) |
|  |  | Enables proactive interventions and optimisation of patient flow through integration of early clinical assessment. (n=6) | *“… this sort of assistance could certainly help direct resources, the right resources, to the right patient, at the right time...”* (P9, Nurse, Junior, Paediatric) |
|  |  | Prompts checks that can enhance patient safety. (n=5) | “…*another set of eyes and ears in an environment where you have a lot of cognitive overload… telling you things that may not necessarily be obvious..*.” (P13, Nurse, Senior, Adult) |
|  |  | Context specific applications used within intended scope. (n=1) | *“If… it has a simple use case which is incredibly important, then everyone will be lining up to get one ...”* (P1, Doctor, Senior, Adult) |
|  | **End user considerations** **(n=8)** | Inexperienced clinicians are provided with a safety net. (n=6) | *“…post-Covid a lot of clinical staff are leaving and they're being replaced with very junior staff, …who just don't have those skills.”* (P12, Nurse, Senior, Adult) |
|  |  | The willingness to embrace AI appears higher among junior clinicians compared to those with extensive clinical experience. (n=2) | *“…I think it could make a big difference for [new] nurses… a lot of them would embrace it.”* (P13, Nurse, Senior, Adult) |
|  | **Adjunct in decision-making** **(n=8)** | Augments, rather than replaces clinician assessments. (n=8) | *“…I would go back to the individual patient data and expand the characteristics based on the alert signals.”* (P14, Doctor, Senior, Neonatal) |
| **Clinician concerns and limitations** | **Over-automation/ over-reliance (n=6)** | May lead to misdirection and impact clinician physical assessment skill due to over-reliance. (n=4) | *“it's got to be accompanied with some really clear support so that clinicians don't just become an instrument of technology …* (P10, Nurse, Senior, Adult) |
|  |  | Less experienced clinicians might overly rely on AI-driven system. (n=2) | *“… I'm sure that there's some junior doctors or … a grad nurse [who] would say I don't care - just tell me what to do.”* (P3, Doctor, Mid, Adult) |
|  | **Accuracy/ false alarms (n=7)** | Frequent and irrelevant/incorrect alerts may contribute to alert fatigue. (n=6) | *“…I think if it gave us too many alerts we wouldn't trust it,”* (P3, Doctor, Mid, Adult) |
|  |  | Alerts and information without context may undermine accurate clinical judgments. (n=1) | *“So if I already know what's going on and you have something that tells me it isn’t there, that will undermine my confidence in what I’m about to do.”* (P1, Doctor, Senior, Adult) |
|  | **Limitations** **(n=9)** | Rigid deterministic solutions that fail to capture the nuances of complex clinical situations. (n=4) | *“…you don’t want them to say you've got to admit this patient to the ICU because my AI algorithm says they're sick and unwell and you … decline it, they go along pass away and instantly that surgeon or the person has grounds for complaint…”* (P5, Doctor, Junior, Adult) |
|  |  | Clinicians’ cognitive biases, anxiety in high-pressure environments and trust issues with algorithms could hinder decision-making. (n=2) | *“…if you get someone new or visiting and they're really anxious, … because they're in this high pressure environment …, so their anxiety translates into how they assess ..., it's essentially putting a bias on them”* (P11, Nurse, Mid, Paediatric) |
|  |  | AI’s adaptability poses challenges in dynamic systems where clinicians must continually adjust to changes in its behaviour. (n=3) | *“Obviously if the tool is then moving and learning over time itself, that would make that a bit difficult because … you're adjusting to it constantly …”* (P4, Doctor, Mid, Adult) |
|  |  | AI alerts may lead to unnecessary anxiety, and inter-clinician conflict, especially if they predict outcomes that cannot yet be acted upon. (n=3) | *“…the conversations you have with ward staff where they would be anxious and agitated about something that hasn't even happened yet, because this number is telling them that it might happen.”* (P5, Doctor, Junior, Adult) |
| **Implementation considerations** | **Research and Development (n=9)** | Supports acceptance through the provision of evidence-backed data, rigorously tested, peer-reviewed research, and transparency of development. (n=9) | *“[Clinicians] have to see the data behind it [data to train the AI] and if you can’t show them data behind it they just will not accept it, it won’t happen”* (P12, Nurse, Senior, Adult) |
|  | **Governance (n=8)** | Supported through policies, procedures and regulatory frameworks ensure AI systems are safe. (n=5) | *“...for [the process of TGA approval] software would also have had relevant clinical trials to validate what it’s saying and what it’s doing...”* (P14, Doctor, Senior, Neonatal) |
|  |  | Through incorporation of safeguards (override functionality) to prevent misdirection and support clinicians to follow evidence-based practice. (n=3) | *“…the worry some clinicians have is that it forces you to question yourself a bit, and it may direct you in the wrong path…so it's just reconciling those two to the point where there's safety in the system ....”* (P11, Nurse, Mid, Paediatric) |
|  | **Education and training (n=7)** | Ensures understanding of strengths and limitations of AI powered CDS. (n=4) | *“…it's part of understanding the strengths and limitations of the tool. I think we should be doing that.”* (P4, Doctor, Mid, Adult) |
|  |  | Requires effective mentorship to ensure AI tools are used appropriately. (n=3) | *“[We need to] make sure that clinicians come back to and go this is a tool to help me, to prompt me, but ultimately this is what I need to do and if I’m not sure about this then I need to call somebody else in who has more experience to help me with what to do next.”* (P10, Nurse, Senior, Adult) |
|  | **Change management (n=4)** | Enabled by a climate of interest and enthusiasm in AI. (n=4) | *“It sounds really exciting that rather than waiting for deterioration to be recognized that that can be picked up early and highlighted to safety teams to prevent acute deterioration and collapse.”* (P9, Nurse, Junior, Paediatric) |
|  |  | Should include meaningful stakeholder engagement is crucial for overcoming resistance to change. (n=1) | *“…change management of getting anything brought in is so difficult and you need a lot of support to get people to expect change…”* (P12, Nurse, Senior, Adult) |
|  |  | User-friendly, intuitive, requiring minimal effort to use (n=1) | *“The ones that actually get used clinically are the ones that require the least amount of effort ...”* (P5, Doctor, Junior, Adult) |
